# The Effect of Natural Colourants in Woad (*Isatis tinctorum* L.) on the Dyeing Properties of Oak and Poplar Wood

**DOI:** 10.3390/ma18194438

**Published:** 2025-09-23

**Authors:** Nikola Kucharczyk, Jan Szadkowski

**Affiliations:** 1 Faculty of Wood Technology, Warsaw University of Life Sciences—SGGW, 02-787 Warszawa, Poland; 2 Department of Wood Science and Wood Preservation, Institute of Wood Science and Furniture, Warsaw University of Life Sciences—SGGW, 02-776 Warsaw, Poland

**Keywords:** veneer, natural dyes, dyer’s woad, colour change, dyeing

## Abstract

The aim of this study was to investigate the potential of dyer’s woad (*Isatis tinctoria* L.) as a sustainable, plant-based colouring agent for enhancing the visual properties of wood surfaces. Veneers of oak (*Quercus* sp.) and poplar (*Populus* sp.) were used as materials and dyed with aqueous extracts derived from fresh and dried woad leaves. To facilitate dye uptake, the veneer surfaces were pretreated with various mordants: alum, citric acid, acetic acid, and distilled water (as a control). The aim was to assess the impact of both the form of the plant material and the type of mordant on the resulting colour change. Colour modifications were measured using spectrophotometry and subjected to statistical analysis. The results revealed distinct differences between untreated and dyed veneers, as well as among samples treated with different mordants. Furthermore, the effectiveness of the dye varied depending on whether fresh or dried leaves were used. These findings confirm the feasibility of employing *Isatis tinctoria* L. as a natural wood dye and highlight its potential as an eco-friendly alternative to synthetic surface treatments in decorative wood applications.

## 1. Introduction

Wood is widely recognised as a highly valued natural material, not only due to its favourable physical and mechanical properties but also owing to its distinctive aesthetic characteristics. The inherent colour, grain structure, and surface texture of wood play a crucial role in determining its decorative value and visual appeal [1].

These attributes are often species-specific and can vary significantly depending on anatomical features and environmental conditions during growth. As a result, the aesthetic qualities of wood frequently serve as a primary criterion in the selection of raw material for architectural applications, interior design, and furniture manufacturing [2].

Among the most commonly used species, oak (*Quercus* sp.) and poplar (*Populus* sp.) are notable for their contrasting features. Oak is a deciduous ring-porous hardwood found widely across Europe. It is known for its high density, strength, and abrasion resistance, making it ideal for flooring, structural applications, and high-end furniture. Its colour ranges from light to dark brown, and when immersed in water, the heartwood tends to darken to grey or even black tones over time [3].

Poplar, on the other hand, is characterised by rapid growth and resistance to weather conditions and drought, enabling it to be cultivated on plantations. It is also a deciduous tree with a ring structure, but due to its pale, greyish–white colour [4,5], it is characterised by significantly lower aesthetic value. Due to these properties, it is often stained. The light colour allows for the production of various colours of this species, often unavailable in species containing higher levels of tannins and colouring agents.

To improve the aesthetic properties of wood, various methods of colour modification are applied, such as thermal modification [6], acetylation [7], or the use of colouring substances [8]. Colouring plays an essential role in finishing processes, not only enhancing visual appeal but also bringing economic benefits [9]. For centuries, people have been developing chemicals, dyes, or mixtures of compounds that change the colours of objects used by humans and the environment in which they live. Nowadays, some of these compounds are used to stain botanical preparations for the purpose of studying cell structures. Important natural dyes include haematoxylin, aniline derivatives, and compounds that react with tannins contained in wood. Haematoxylin is obtained by extraction from the heartwood of *Haematoxylum campechianum* L. Through the use of an appropriate extraction and oxidation procedure, it can stain wood in shades of black, blue, and even yellow. Metal salts, such as iron, are also used to change the colour of wood. They react with compounds contained in wood, leading to a change in colour. Alkaline compounds, such as sodium compounds, are used to bleach wood [10,11,12]. Another relevant group is vat dyes—water-insoluble dyes that are chemically reduced to a soluble, colourless leuco form, allowing penetration into fibres. Upon re-oxidation, they revert to their insoluble, coloured state, providing durable and stable colouration of the material [13].

In recent years, researchers have increasingly focused on natural dyes, most of which are plant-derived—from roots, bark, wood, and leaves—as environmentally friendly alternatives to synthetic dyes [14,15].

One such plant harvest for natural pigment is dyer’s woad (*Isatis tinctoria* L.), known in Europe as an ancient source of indigo dye. It was used already during the Bronze and Iron Ages [16,17,18]. The plant is easy to cultivate, grows rapidly, and shows resistance to weather conditions and biological factors. Its dual functionality—as a medicinal plant and a dye source—is reflected in its name: *Isatis* refers to healing properties, while *tinctoria* points to its dyeing function [19,20].

Indigoid dyes are based on two indoxyl units and belong to the group of vat dyes. In an alkaline environment with a reducing agent, they transform into a water-soluble, colourless leuco form. Once applied, they oxidise in contact with air and return to their characteristic blue colour [21]. Using these techniques, a wide palette of blue shades can be achieved, from light “watchet” tones to the deep “Bleu d’enfer” of 18th-century France [8].

Indigo obtained from *Isatis tinctoria* is less intense and durable than the indigo from *Indigofera tinctoria* L., also known as “true indigo”, but it contains a wider variety of organic colouring compounds, including indigotin and indirubin [22]. Indoxyl is a key reactive precursor (3-hydroxyindole) derived from indican in plants, which undergoes oxidative dimerisation to form indigoid dyes. The most prominent product, indigotin (indigo), is a blue, water-insoluble dye extensively used as a vat dye due to its durability on fibres. In smaller amounts, indirubin, a red structural isomer of indigotin, is also produced during the oxidation of indoxyl, contributing to the range of natural hues obtained from indigo-containing plants [17,19,20,23,24].

Unlike standard indigo dyeing, dyeing with fresh leaves relies on fermentation in water, where precursors convert to indoxyl. An alkaline agent is used to maintain the required pH and neutralise acids formed during fermentation. Although there is no documentation of this method for woad specifically, it is likely to have existed due to its simplicity and early use in other dye plants [24,25,26].

Indigo and imperial purple belong to the same chemical group. Both dyes share similar properties and undergo the same transformation mechanisms during dyeing [18].

Also, recent studies have explored other plant-based dyes for wood colouration and protection. For instance, Dalbergia extracts have been shown to impart stable reddish–brown hues while providing moderate resistance to photodegradation [27,28]. Turmeric-based dyes, on the other hand, offer bright yellow tones but are generally less stable under prolonged light exposure [29,30].

Before dyeing with natural dyes, fabric fibres were activated using substances such as weak organic acids (approximately 2% acetic acid), alkaline compounds (aqueous ammonia solution, sodium hydroxide), and hydrogen peroxide [31,32]. The activation of fabrics was intended to damage the fibres of the materials in order to better penetrate the pigments into the material, which enabled greater and more durable dyeing of the material [33,34]. This process also affects the wood’s colour and the intensity of the resulting stain. In the case of wood, the use of colour activators is more difficult due to the complex chemical structure of wood and the interweaving of raw materials such as cellulose, lignin, hemicelluloses, and extractives that affect the wood’s colour [32,34,35].

Woad (*Isatis tinctoria* L.) has been used for centuries as a natural source of indigo dye, with a rich history of application in textile colouration. The chemical properties, extraction methods, and dyeing techniques for fibres such as wool and linen are extensively documented. In contrast, the use of woad for staining or colouring wood remains largely unexplored, and there is a lack of systematic studies evaluating its efficacy, stability, and interaction with lignocellulosic materials. There is also a lack of methodology for colouring wood surfaces using natural pigments and plant extracts. This leads to the need to develop a methodology for preparing the surface for colouring as well as the colouring procedure itself to ensure colour repeatability on the coloured surface.

The objective of this study was to evaluate the effect of a vegetable dye extracted from *Isatis tinctoria* L. on the colour of oak and poplar veneers. Additionally, the study emphasised the significance of selecting the appropriate combination of plant form and mordant to achieve the desired colour intensity and uniformity.

## 2. Materials and Methods

### 2.1. Materials

The test material consisted of oak and poplar veneers. Sheets measuring 90 × 70 × 0.6 mm for poplar and 135 × 70 × 0.6 mm for oak were prepared for testing. The veneers used in the study originated from the same bundle and were plain-sliced, ensuring that all samples had the same anatomical orientation.

Woad was used as the colouring agent. The woad leaves were collected from a location near the Vistula River in Warsaw (52°16′49″ N, 20°59′37″ E). The leaves were harvested from first-year plants, which are considered optimal for dye extraction. The collected material was divided into two equal portions. The first portion was retained in its original form (whole leaves), while the second portion was blended and then dried in an oven at 75 °C for 24 h.

After drying, the moisture content of the blended woad was determined using the oven-drying method. The moisture content was found to be 7.76%.

A dye solution from woad (*Isatis tinctorum* L.) for variants with fresh and dried leaves was obtained for fresh leaves by weighing 150 g and adding 200 mL of distilled water, and for dried leaves by weighing 3.75 g and adding 150 mL of distilled water. The prepared solutions were placed in a laboratory water bath (manufactured by World Science Laboratory—WSL, Świętochowice, Poland) to prevent the mixture from overheating, at a water temperature of 80 °C for 10 h. The solutions obtained in this way were filtered using a metal laboratory sieve with a mesh diameter of 0.08 mm.

### 2.2. Surface Mordanting

To verify the repeatability of the colouration, the prepared materials were placed four at a time in crystallisers with activators. The crystallisers were then placed in batches into the oven and held for one hour at 95 °C. After removal from the oven, the materials were removed from the solutions, surface-dried with a paper towel, and placed in the dryer for 15 min at 75 °C.

Mordants used to activate the surface for staining:

- distilled water (control activator),
- 2% alum (AlK(SO_4_)_2_·12H_2_O),
- 2% citric acid solution (C_6_H_8_O_7_·H_2_O),
- 3% vinegar solution (CH_3_COOH).

### 2.3. Colouring

The resulting extracts were transferred into crystallisers, and 15 mL of 25% ammonia solution was added per 100 mL of extract. Following mixing, the solutions were allowed to stand for 10 min. After this period, the activated veneers were introduced into the extracts in batches and placed in a drying oven for 1 h at 55 °C. Subsequently, the veneers were removed, surface-dried with a paper towel, and then returned to the oven for 15 min at 75 °C.

### 2.4. Colour Measurement

The colour measurements were performed using a spectrophotometer, specifically the ERICHSEN SPECTROMASTER Model 565-D (Iserlohn, Germany). This instrument permits the measurement of colour through the use of three colour indices: L*, a*, and b*. The L* index represents the colour range from darkness to lightness, and the a* value indicates the position on the red–green axis, with positive values indicating red and negative values indicating green. The b* value represents the position on the yellow–blue axis, with positive values indicating yellow and negative values indicating blue.

Colour differences were assessed by comparing these indices, and the overall colour change (ΔE*) was calculated using the following formula:

(1) $$∆E=\sqrt{{\left(∆L\right)}^{2}+{\left(∆a\right)}^{2}+{\left(∆b\right)}^{2}}$$

The parameters ΔL, Δa, and Δb represent the changes in colour coordinates in the CIELAB colour space. Specifically, ΔL indicates the change in lightness, Δa the change along the red–green axis, and Δb the change along the yellow–blue axis. These values are calculated as the difference between the measured values of the dyed sample and the untreated (control) sample:

(2) $$∆L=\;L_{\mathrm{dyed}}-L_{\mathrm{control}}\;∆a=\;a_{\mathrm{dyed}}-a_{\mathrm{control}}\;∆b=\;b_{\mathrm{dyed}}-b_{\mathrm{control}}$$

**Figure 1:**
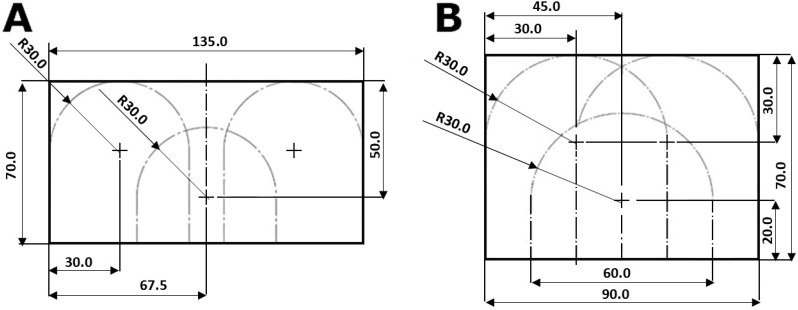
Measuring points for poplar (**A**) and oak (**B**) veneers (Units: mm).

The colour of oak and poplar veneer sheets was measured at three predefined points, following the layout shown in Figure 1. A minimum of nine measurements per sheet ensured repeatability and reliability. This method reduced the effect of natural variations in texture and grain, providing data that reflected the overall colour characteristics of the material rather than local irregularities.

### 2.5. Statistical Analysis

Statistical analysis of the obtained changes in the L*, a*, and b* indices responsible for mathematical colour mapping was carried out on Statistica 13 (TIBCO, Palo Alto, CA, USA) Software Inc. (2017). The multivariate analysis of variance (ANOVA) was used to test (α = 0.05) for significant differences between factors. A comparison of the group means was conducted using the Tukey post hoc test, which allowed for the identification of specific differences between groups. Statistical significance level was 0.05.

## 3. Results

**Figure 2:**
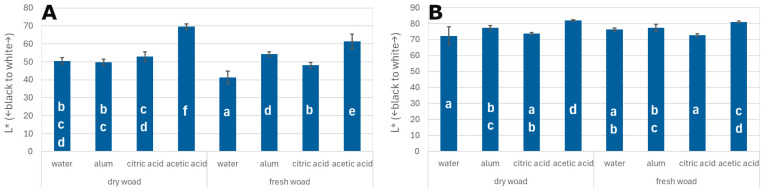
L* index for oak veneers (**A**) and poplar veneers (**B**); different letters—homogenous group by Tukey test (α = 0.05).

The results of the colour measurements of oak veneers dyed with woad are presented in Figure 2A. A detailed analysis of the data indicates that the highest lightness (L*) value was recorded for veneers dyed with dried woad leaves using an acetic acid mordant (L* = 70). For samples dyed with fresh woad leaves and acetic acid mordant, the L* value was 60. The L* values obtained for veneers treated with acetic acid mordant were statistically significantly higher than those for other dyeing variants. The lowest L* value was observed in veneers treated with distilled water mordant and dyed with fresh woad leaves, with an L* value of 40.

In the case of poplar veneers, the results presented in Figure 2B indicate that the highest lightness (L*) value was observed for veneers dyed with dried woad leaves using an acetic acid mordant (approximately L* = 82). However, the data show no statistically significant differences in lightness values between the various mordants used. Therefore, it can be concluded that the type of mordant did not have a substantial effect on the variation in lightness.

**Figure 3:**
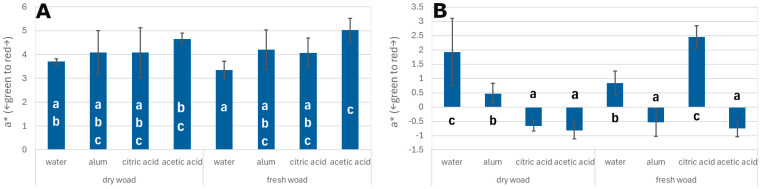
a* index for oak veneers (**A**) and poplar veneers (**B**); different letters—homogenous group by Tukey test (α = 0.05).

When analysing the results for the green–red colour axis (a*), oak veneers treated with acetic acid again exhibited the highest a* values. For dyeing with dried woad leaves, the a* value was approximately 4.7, while for dyeing with fresh leaves it reached 5.0. The lowest a* value within this range was recorded for veneers treated with distilled water mordant and dyed with fresh leaves (a* = 3.2) (Figure 3A).

In the case of poplar veneers, the highest a* value was observed for samples treated with acetic acid and dyed with fresh woad leaves (a* = 2.5) (Figure 3B). Conversely, when fresh leaves were used and the veneers were treated with distilled water, the a* value was negative, at approximately −0.8. For dyeing with dried woad leaves, the highest a* value was found in veneers treated with distilled water, with a value of approximately 1.9.

**Figure 4:**
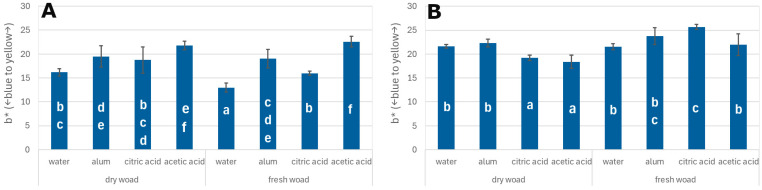
b* index for oak veneers (**A**) and poplar veneers (**B**); different letters—homogenous group by Tukey test (α = 0.05).

An analysis of the results shows that the highest value on the blue–yellow colour axis (b*) for oak veneers was recorded in samples dyed with fresh woad leaves using an acetic acid mordant (approximately b* = 23). When dried leaves were used with acetic acid mordant, the b* value was slightly lower, at around 22. The b* values obtained with acetic acid mordant were statistically significantly higher than those recorded for other dyeing variants. The lowest b* value was observed in veneers treated with distilled water mordant and dyed with fresh woad solution, where the value was approximately 13 (Figure 4). This group showed the strongest shift towards the blue region of the spectrum.

In the case of poplar veneers, the highest b* value was also observed in samples dyed with fresh woad leaves using acetic acid mordant, reaching approximately 26. In contrast, staining with dried leaves and the same mordant resulted in a b* value of around 18, which was the lowest value among the poplar samples and indicated the greatest shift towards blue.

The analysis of variance showed that for the lightness parameter (L*), both the woad leaf preparation method (fresh or dried) and the type of mordant had a statistically significant effect (Table 1). The mordant was the main influencing factor, with percentage influence coefficients of 75.59% for oak veneer and 65.38% for poplar veneer. The interaction between leaf preparation and mordant was significant for oak (10.46%) but lower for poplar (6.67%), and a high residual error of 27.62% for poplar suggests other unexamined factors may also contribute.

For the green-to-red parameter (a*), no significant effects were observed for oak veneer. The mordant contributed 36.68%, while the residual error was high (60.41%), indicating other factors likely influenced the results. The interaction between preparation and mordant was minimal (2.87%). In contrast, for poplar veneer, both factors significantly affected a* values, with mordant contributing 41.39% and the interaction between preparation and mordant contributing 43.34% (Table 2).

Regarding the blue-to-yellow parameter (b*), both factors significantly influenced measurements. For oak veneer, mordant was dominant (70.21%), with a residual error of 18.97%. For poplar veneer, the preparation method contributed 34.34%, and the interaction with mordant was significant. A residual error of 20.88% suggests additional unaccounted factors.

As presented in Table 3, the colour differences (ΔL, Δa, Δb) in oak and poplar veneers dyed with fresh and dry woad leaves using various mordants as well as total colour difference (ΔE), are shown. A detailed analysis of the table reveals that the most significant colour change (ΔE = 27.24) occurred in veneers treated with fresh woad leaves and water used as a mordant. In contrast, for veneers dyed with dry woad leaves, the highest colour difference was observed in samples treated with alum mordant, where ΔE reached a value of 19.

Table 3, which presents data for poplar veneer, shows that the greatest colour change (ΔE = 16.13) occurred in veneers dyed with fresh woad leaves using citric acid as a mordant. Conversely, when dry woad leaves were used in combination with distilled water as the mordant, the colour difference decreased to ΔE = 12.17.

The lowest colour change for oak veneer was observed in samples dyed with both types of woad leaf preparations (fresh and dry) using acetic acid as a mordant. In this case, ΔE was 1.99 for dry leaves and 9.19 for fresh leaves. For poplar veneer, the lowest colour change was recorded in veneers stained with dry woad leaves and treated with acetic acid, where ΔE was approximately 4.44.

The values of ΔL show that most treatments resulted in a substantial decrease in lightness, particularly in oak veneers, where the ΔL dropped as much as −26.32. In contrast, poplar veneers showed more moderate changes in lightness. The Δa and Δb components also varied depending on the wood species and treatment, reflecting shifts along the red–green and yellow–blue axes, respectively.

Overall, the results demonstrate that both the form of woad leaves (fresh or dry) and the type of mordant significantly affect the colour outcome of the dyeing process, with fresh woad combined with water or citric acid producing the most intense colour changes.

**Figure 5:**
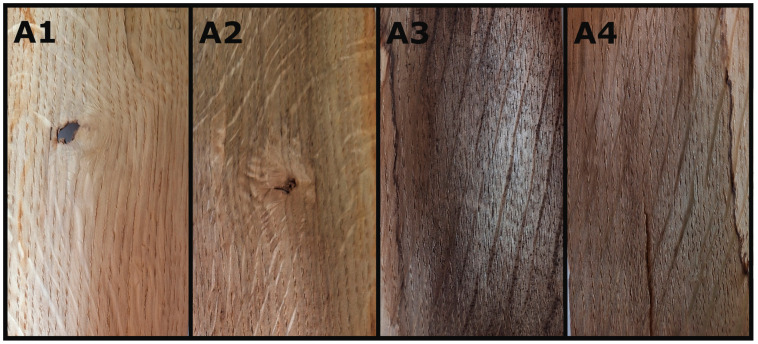
Colour change observed on oak veneers: **A1**—clear, **A2**—after mordant, **A3**—dyed with dried leaves, **A4**—dyed with fresh leaves.

**Figure 6:**
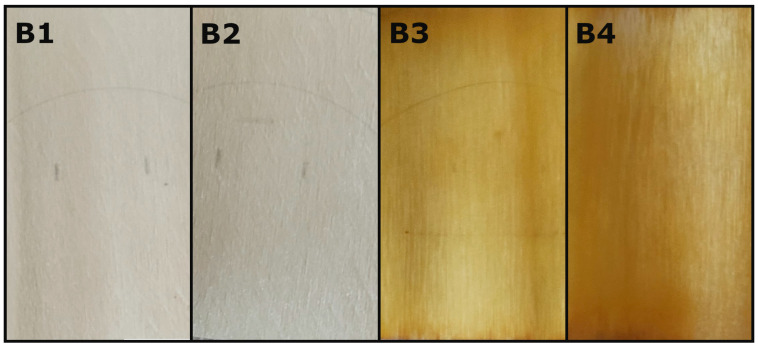
Colour change observed on poplar veneers: **B1**—clear, **B2**—after mordant, **B3**—dyed with dried leaves, **B4**—dyed with fresh leaves.

Figure 5 and Figure 6 show the colour change where A/B1 shows the veneer before any chemical preparation, A/B2 the veneer after mordant (distilled water), A/B3 the veneer dyed with dried leaves, and A/B4 the veneer dyed with fresh leaves.

Measurement data from the experiments performed are included in the Supplementary Materials.

## 4. Discussion

The conducted study confirmed that both the preparation method of *Isatis tinctoria* L. and the type of mordant significantly influenced the final colour change of oak and poplar veneers. These findings are consistent with those reported by Vauquelin et al. [36], who demonstrated that the extraction conditions and oxidative medium used in the processing of woad have a substantial effect on the dye yield and chromatic outcome.

The most intense modification was observed in oak veneers dyed with fresh woad leaves activated using distilled water, while the least pronounced change occurred in samples treated with acetic acid and dyed with dried leaves. For poplar veneers, the strongest colour effect was achieved using fresh leaves combined with citric acid, whereas the weakest effect was again observed for dried leaves paired with acetic acid.

The higher tannin content in oak can enhance dye uptake by providing additional binding sites for the woad pigments, which may contribute to the stronger influence of mordant observed in oak veneer. In contrast, the lower extractive content in poplar could reduce its interaction with the dye, making the preparation method and the interaction with mordant more critical for colour development [37,38].

These findings are consistent with previous research demonstrating that dye uptake and fixation strongly depend on both the freshness of the botanical material and the chemical nature of the mordant, which modulate molecular interactions with wood substrates [39]. Although studies specifically focusing on the preparation of *Isatis tinctoria* leaves remain limited, the overall trends observed here correspond to broader patterns reported for plant-based dyes applied to lignocellulosic materials.

Moreover, our results underscore the importance of optimising dyeing protocols—particularly the selection of the appropriate combination of plant form and mordant—to achieve the desired colour intensity and uniformity. This aligns with previous observations indicating that natural dye systems require iterative application and careful parameter control to maximise performance and consistency across substrates [10,12]. Weigl and colleagues highlighted the multifactorial complexity of colour alterations in wood treated with natural extracts, while Zhu et al. [40] demonstrated that the deposition of plant pigments can enhance not only aesthetics but also the photoprotection of wood, particularly against UV-induced degradation.

Furthermore, the results obtained in this study for wood veneers dyed with naturally sourced indigo are in line with other reports on natural dyes. It was demonstrated that the extent of colour change is influenced by both the sample preparation method (e.g., fresh or dry plant material) and the wood species. These findings support the conclusions of Vespignani et al. [12], who emphasised the importance of conducting multiple dyeing cycles to fully evaluate the performance of natural colourants.

It is well established that wood and wood-based products exhibit inherent variability in colour, which can be further modified through dyeing processes. The use of natural dyes in this context is particularly relevant, offering not only aesthetic benefits but also aligning with the principles of a sustainable and circular economy. This is especially important for the wood industry, which increasingly seeks environmentally friendly surface treatments.

Although the mechanisms governing colour modification in wood are complex and influenced by various physical and chemical factors, the incorporation of natural dyes plays a critical role in this transformation [10]. Additionally, as highlighted by Zhu et al. [40], the deposition of plant-based pigments on wood fibres is not only effective in achieving colouration but may also enhance surface protection, particularly against UV radiation. These combined effects suggest that natural dyeing can contribute to both the visual and functional enhancement of wood products.

## 5. Conclusions

The results confirmed that both the preparation method of the *Isatis tinctoria* L. extract and the type of mordant had a significant effect on the colouring of oak and poplar veneers. The most intense colour change was observed in oak veneers dyed with fresh leaves and distilled water and in poplar veneers treated with fresh leaves and citric acid. Statistical analysis indicated that the mordant exerted the greatest influence on the colour parameters, particularly within the black-to-white (∆L) and blue-to-yellow (∆b) ranges. These findings demonstrate the potential of dyer’s woad as a sustainable natural dye for wood surfaces and underscore the need for further research on process optimisation and the long-term durability of the obtained colour effects.

## Figures and Tables

**Table 1:** Analysis of variants of studied factors influencing staining on oak veneers.

	L		a		b	
	*p*	X	*p*	X	*p*	X
woad	0.000	6.96	0.870	0.04	0.000	4.56
mordant	0.000	75.59	0.000	36.68	0.000	70.21
woad x mordant	0.000	10.46	0.597	2.87	0.009	6.26
Error		6.99		60.41		18.97

*p*—significant with α = 0.05; X—percentage of contribution.

**Table 2:** Analysis of variants of studied factors influencing staining on poplar veneers.

	L		a		b	
	*p*	X	*p*	X	*p*	X
woad	0.497	0.33	0.084	1.11	0.000	34.34
mordant	0.000	65.38	0.000	41.39	0.000	19.56
woad x mordant	0.033	6.67	0.000	43.34	0.000	25.22
Error		27.62		14.17		20.88

*p*—significant with α = 0.05; X—percentage of contribution.

**Table 3:** Colour Differences (ΔL, Δa, Δb, ΔE) in oak and poplar veneers dyed with fresh and dry woad leaves using various mordants.

Wood	Variant of Woad Leaf	Mordant	∆L	∆a	∆b	∆E
Oak	Dry woad	water	−17.52	−2.47	−7.65	18.13
		alum	−17.86	−1.96	−4.52	18.74
		citric acid	−15.87	−1.73	−2.92	16.00
		acetic acid	−0.60	−1.59	−1.13	1.99
	Fresh woad	water	−26.32	−2.65	−10.11	27.24
		alum	−15.47	−1.84	−3.34	16.20
		citric acid	−21.82	−1.70	−5.76	22.24
		acetic acid	−9.10	−1.04	−0.24	9.19
Poplar	Dry woad	water	−10.63	3.32	5.32	12.17
		alum	−6.18	2.02	6.45	7.88
		citric acid	−10.53	1.00	3.80	10.85
		acetic acid	−3.53	0.67	2.96	4.44
	Fresh woad	water	−7.79	2.59	6.44	10.58
		alum	−6.44	1.00	8.27	10.46
		citric acid	−12.96	4.03	9.86	16.13
		acetic acid	−4.79	0.75	6.37	8.02

## Data Availability

The original contributions presented in this study are included in the article/Supplementary Materials. Further enquiries can be directed to the corresponding authors.

## Supplementary Materials

The following supporting information can be downloaded at: https://www.mdpi.com/article/10.3390/ma18194438/s1. Excel sheet with measurement data.
